# Telmisartan Attenuates Kanamycin-Induced Ototoxicity in Rats

**DOI:** 10.3390/ijms222312716

**Published:** 2021-11-24

**Authors:** Chang Ho Lee, So Min Lee, So Young Kim

**Affiliations:** Department of Otorhinolaryngology-Head & Neck Surgery, CHA University College of Medicine, Seongnam 13496, Korea; hearwell@gmail.com (C.H.L.); lws6812@naver.com (S.M.L.)

**Keywords:** telmisartan, aminoglycosides, hearing loss, peroxisome proliferator activator receptor-γ, angiotensin II receptor blocker

## Abstract

Telmisartan (TM) has been proposed to relieve inflammatory responses by modulating peroxisome proliferator activator receptor-γ (PPARγ) signaling. This study aimed to investigate the protective effects of TM on kanamycin(KM)-induced ototoxicity in rats. Forty-eight, 8-week-old female Sprague Dawley rats were divided into four groups: (1) control group, (2) TM group, (3) KM group, and (4) TM + KM group. Auditory brainstem response was measured. The histology of the cochlea was examined. The protein expression levels of angiotensin-converting enzyme 2 (ACE2), HO1, and PPARγ were measured by Western blotting. The auditory threshold shifts at 4, 8, 16, and 32 kHz were lower in the TM + KM group than in the KM group (all *p* < 0.05). The loss of cochlear outer hair cells and spiral ganglial cells was lower in the TM + KM group than in the KM group. The protein expression levels of ACE2, PPARγ, and HO1 were higher in the KM group than in the control group (all *p* < 0.05). The TM + KM group showed lower expression levels of PPARγ and HO1 than the KM group.TM protected the cochlea from KM-induced injuries in rats. TM preserved hearing levels and attenuated the increase in PPARγ and HO1 expression levels in KM-exposed rat cochleae.

## 1. Introduction

Telmisartan (TM) is an angiotensin II (AngII) receptor blocker [1,2]. TM selectively inhibits AngII type 1 receptor (AT1R) [1]. AT1R activates several signal transduction pathways, including G-protein-mediated activation of nuclear factor-κB (NF-κB) and intracellular kinases, such as mitogen-activated protein kinase (MAPK) and Akt/protein kinase B [3]. Moreover, AT1R activation generates reactive oxygen species (ROS) and induces small GTPases [3,4]. As an AT1R antagonist, TM decreases the angiotensin-converting enzyme (ACE)/AngII/AT1 axis and increases ACE2/Ang(1-7)/Mas axis activation [5]. The activation of ACE2 and subsequent induction of Ang(1-7) and Mas have been reported to have anti-inflammatory, antifibrotic, and antithrombotic actions [6,7]. Through the modulation of the renin–angiotensin system to favor the ACE2/Ang(1-7)/Mas axis, TM demonstrated therapeutic effects for a number of disorders, including nonalcoholic fatty liver disease and metabolic syndrome [5,8]. In addition, recent studies suggested that the antagonizing action for the production of AngII and anti-inflammatory responses of TM could exert therapeutic effects on COVID-19 [9].

In addition to being an AT1R blocker, TM has been acknowledged as a partial agonist of peroxisome proliferator activator receptor-γ (PPARγ) [1,10]. PPARγ plays a role in maintaining the homeostasis of lipid and glucose metabolism and inflammatory cells [11,12]. PPARγ inhibits inflammatory responses via suppression of a number of signaling pathways, such as the NF-κB and MAPK pathways [11]. Aminoglycoside is commonly prescribed to treat bacterial infections, including multidrug resistant turberculosis. In patients who were prescribed aminoglycosides, as many as 36.7% of patients were reported to have aminoglycoside-induced ototoxicity [13]. Oxidative stress and inflammatory responses are the pathophysiological mechanisms of aminoglycoside-induced ototoxicity [14]. Aminoglycosides induce high calcium uptake by mitochondria in cochlear hair cells, which results in the secretion of pro-apoptotic factors and oxidative enzymes into the cytoplasm and generates reactive oxidative species [15]. The attenuation of oxidative stress and inflammatory responses was reported to have a protective effect on aminoglycoside-inducted ototoxicity [16]. Therefore, TM was presumed to have a protective effect on aminoglycoside-induced hearing loss. An in vitro study described the protection of cochlear hair cells from gentamicin-induced ototoxicity by co-treatment with TM in cochlear explant models [17]. The otoprotective effect of TM was completely blocked when exposed to the irreversible PPARγ antagonist GW9662, in that the authors supposed that partial agonism of PPARγ mediates the otoprotective effect of TM in cochlear explant models [17]. However, to the best of our knowledge, the otoprotective effects of TM have not been evaluated in animal models.

We hypothesized that TM could suppress kanamycin (KM)-induced ototoxicity in an in vivo model. This study was aimed to investigate the effect of TM on KM-induced toxicity in an in vivo model. Antioxidative responses involving PPARγ may diminish the oxidative stress response in KM-induced ototoxicity. In addition, it was speculated that modulation of the renin–angiotensin system could be accompanied by otoprotective effects of TM. To test these effects, TM was administered to rats with or without KM in rat models. Auditory function and cochlear histology were compared to examine the effect of TM administration on KM-induced ototoxicity. The expression levels of the oxidative stress markers HO1, PPARγ, and ACE2 were evaluated in TM + KM rats and compared with those in KM and control rats.

## 2. Results

**Figure 1:**
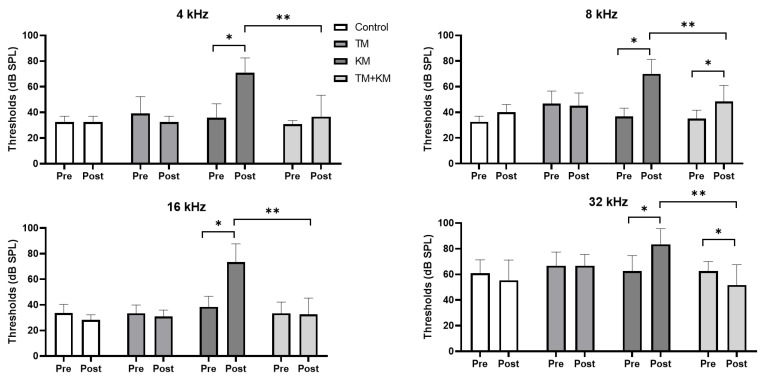
Auditory brainstem response (ABR) thresholds at 4, 8, 16, and 32 kHz pre- and post-drug administrations. TM: flutamide, KM: kanamycin, * *p* < 0.05 paired *t*-test between pre- and post-treatment ABR thresholds, ** *p* < 0.05 in unpaired *t*-test between KM and KM + TM groups.

The auditory thresholds did not change after TM treatment (Figure 1). Following KM administration, the auditory thresholds were elevated at 4, 8, 16, and 32 kHz (all *p* < 0.05). The rats co-treated with TM + KM showed attenuated threshold shifts at 4, 8, 16, and 32 kHz compared with those of KM rats (all *p* < 0.05). The average auditory threshold was 36.66 dB SPL (SD = 16.69) vs. 70.83 dB SPL (SD = 11.64) at 4 kHz, 48.33 dB SPL (SD = 12.67) vs. 70.00 dB SPL (SD = 11.28) at 8 kHz, 32.50 dB SPL (SD = 12.88) vs. 73.33 dB SPL (SD = 14.35) at 16 kHz, and 51.66 dB SPL (SD = 15.85) vs. 62.50 dB SPL (SD = 7.53) at 32 kHz for the post-TM + KM administration (*n* = 12) and post-KM treatment groups (*n* = 12).

**Figure 2:**
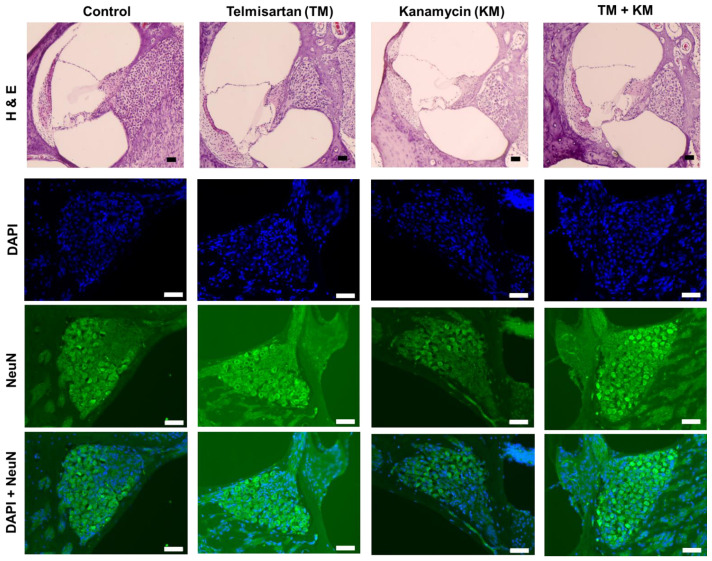
Hematoxylin and eosin (H&E) staining of the cochlea and immunofluorescence staining (Green: NeuN-positive cells; Blue: DAPI-positive cells). The KM + KM group showed smaller changes in the loss of spiral ganglion cells than the KM group, scale bar: 50 µm.

**Figure 3:**
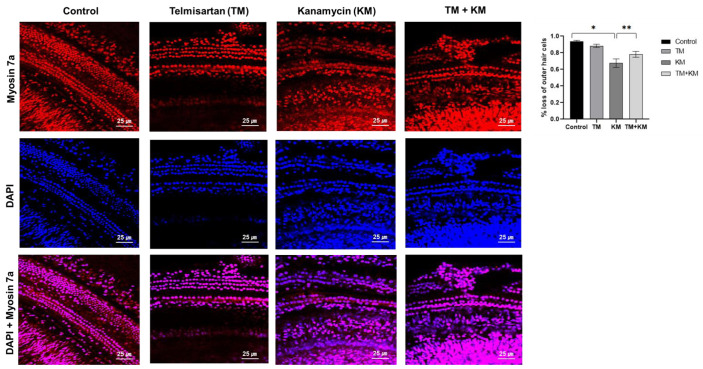
The cochlear whole-mount (Red: myosin7A-positive cells; Blue: DAPI-positive cells; Purple: myosin7A and DAPI-positive cells). The KM + KM group showed smaller changes in the loss of outer hair cells than the KM group. * *p* < 0.05 unpaired *t*-test between THE control and KM groups, ** *p* < 0.05 in unpaired *t*-test between KM and KM + TM groups.

Histologic examination of the cochleae demonstrated the loss of outer hair cells and spiral ganglial cells in KM rats (Figure 2 and Figure 3). Compared with KM rats, TM + KM rats showed preserved cochlear outer hair cells and spiral ganglial cells. H&E staining and immunostaining with NeuN showed partial loss of spiral ganglial cells in KM rats, which were preserved in TM + KM rats (Figure 2). The outer hair cells were disoriented and partially missed in KM rats, which were attenuated in TM + KM rats (Figure 3).

**Figure 4:**
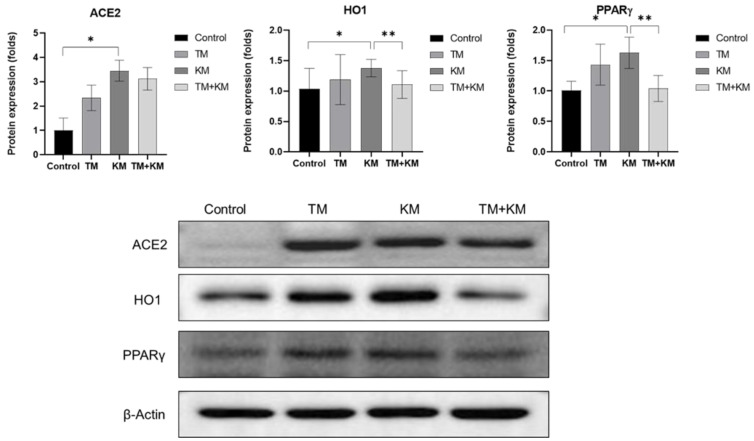
The protein expression levels of angiotensin converting enzyme 2 (ACE2), heme oxygenase 1 (HO1), and peroxisome proliferator activator receptor-γ (PPARγ). The KM + TM group showed lower levels of HO1 and PPARγ than the KM group (* *p* < 0.05 in unpaired *t*-test between control and KM groups, ** *p* < 0.05 in unpaired *t*-test between KM and KM + TM groups).

To explore the related molecular mechanisms, the cochlear protein expression levels of ACE2, HO1, and PPARγ were estimated using Western blotting (Figure 4). TM rats did not show significant differences in the protein expression levels of ACE2, HO1, and PPARγ in control rats. The protein expression level of ACE2 increased in KM rats compared with that in control rats (1.00 ± 0.51 vs. 3.45 ± 0.43, *p* < 0.001). The protein expression level of ACE2 was not significantly different between KM and TM + KM rats (3.45 ± 0.43 vs. 3.12 ± 0.45, *p* = 0.226). The protein expression level of HO1 was higher in KM rats than in control rats (1.38 ± 0.14 vs. 1.03 ± 0.33, *p* = 0.046). Compared with KM rats, TM + KM rats showed a lower expression level of HO1 (1.38 ± 0.14 vs. 1.10 ± 0.22, *p* = 0.034). The expression level of PPARγ was higher in KM rats than in control rats (1.01 ± 0.14 vs. 1.63 ± 0.25, *p* < 0.001). TM + KM rats demonstrated a lower expression level of PPARγ than KM rats (1.04 ± 0.21 vs. 1.63 ± 0.25, *p* = 0.002).

## 3. Discussion

TM administration attenuates KM-induced hearing loss in rat models. The TM co-treated rats demonstrated preserved cochlear outer hair cells and spiral ganglial cells. The otoprotective effect of TM was accompanied by a decrease in oxidative stress-related protein levels of HO1 and PPARγ, which implied the mediating roles of antioxidative responses. This is a novel study on the protective effect of TM on aminoglycoside-induced ototoxicity in vivo.

Aminoglycosides have been shown to induce injuries to auditory hair cells and spiral ganglial cells by promoting oxidative responses and inflammation [18]. In addition to outer hair cells and spiral ganglial cells, various regions of the cochlea—such as presynaptic ribbon synapses and stria vascularis—are involved in aminoglycoside-induced ototoxicity [19,20]. Cochlear inner hair cell ribbon synapses were reported to be more susceptible to aminoglycoside-induced ototoxicity and related to low-dose aminoglycoside-induced ototoxicity without loss of outer hair cells and spiral ganglial cells [19]. Moreover, aminoglycoside-induced cochlear injuries can be potentiated by systemic inflammation via vasodilation of capillaries in the stria vascularis [20]. Thus, one of the therapeutic strategies against aminoglycoside-induced ototoxicity has targeted inflammatory responses and oxidative stresses in the cochlea [21]. A number of antioxidant molecules—such as dihydromyricetin, nuclear factor erythroid-2-related factor 2, and avocado oil extract—have been reported to have protective effects against oxidative stress and inflammatory conditions and aminoglycoside-induced ototoxicity [22,23,24,25,26,27]. However, few therapeutic agents are clinically available for aminoglycoside-induced ototoxicity. The repositioning of drugs used for other diseases could facilitate the clinical application of drugs for aminoglycoside-induced ototoxicity in cost-effective and effort-effective ways. TM is a widely used antihypertensive drug that can be used to protect against hearing loss in patients with aminoglycoside treatment.

The modulation of PPARγ was observed in TM-treated rats in the present study. In line with this, several previous studies have reported the otoprotective roles of PPARs [28,29,30]. A cochlear explant study demonstrated the detoxification of ROS and protection of hair cells from gentamicin-induced toxicity by the PPARγ agonist pioglitazone [28]. In cochlear explant and rat models of gentamicin-induced ototoxicity, fenofibrate and PPARα agonists exert otoprotective effects and reduce ROS by the HO-1 signaling pathway [29]. In addition to aminoglycoside-induced toxicity, the agonism of PPARγ using pioglitazone protected against noise-induced hearing loss in a rat model [30]. Intratympanic injection of pioglitazone suppressed inflammatory and oxidative responses by attenuating the expression of NF-κB and interleukin (IL)-1β in the cochlea [30]. PPARγ is a ligand-activated transcription factor that activates anti-inflammatory cascades through HO-dependent signaling [31]. HO1 has been reported to activate macrophages and reduce neutrophil responses [31]. The increased level of HO1 in KM rats implied a reactive anti-inflammatory response following KM ototoxicity. In line with this, previous studies also demonstrated increased levels of antioxidants, such as PPARγ, HO1, and superoxide dismutase 1, following ototoxic drug exposure in rats [29,32]. TM rats showed an increased tendency of PPARγ and HO1 expression in the present study. However, the variations in expression levels were considerable and the difference was not significant compared with control rats. Blocking the AT receptor could augment the anti-inflammatory responses and increase the anti-inflammatory molecules PPARγ and HO1.

The expression level of ACE2 was high in KM rats, and TM treatment did not reverse the increased ACE2 levels in this study. TM rats showed a tendency of increased ACE2 expression which did not demonstrate the significant difference with KM rats in this study. The augmented expression of ACE2 following TM administration could be explained by the inhibition of the AT1 receptor and the activation of the ACE2/Ang(1-7)/Mas axis [5]. TM was reported to increase ACE2 activation, and ACE2-mediated generation of Ang(1-7) is linked with anti-inflammatory functions [6,33]. Thus, it can be supposed that the activation of ACE2 signaling by TM treatment may maintain the increased level of ACE2 in TM + KM rats in this study. ACE2 expression was elevated in KM rats in this study. The ACE2 elevation in KM rats may be a compensatory mechanism to cope with aminoglycoside-induced ototoxicity. Although there has been no prior research on ACE2 expression following ototoxic insults, ACE2 activation has protective effects in gentamicin-induced nephrotoxicity rat models by increasing Ang(1-7) while reducing inflammatory markers, such as TNFα, IL-6, and NF-κB [34,35]. In addition, TM administration improved cognitive function in an Alzheimer rat model with activation of ACE2 and Ang(1-7) [33]. To delineate the effect of TM on ACE2/Ang(1-7)/Mas axis activation, examination of the changes in Ang (1-7), Mas, and related biological markers may be warranted. In addition, the opposing effects on the ACE1/AngII/AT1 axis need to be explored. Although we examined cochlear morphology using H&E, immunofluorescence staining, and cochlear whole mounts, the small number of examined cochleae and limited resolution of immunostaining restricted the quantification of outer hair cell and spiral ganglial cell injuries. Further studies using long-term and/or other doses of drug administration with a larger number of study groups need to be conducted to optimize the otoprotective effects of TM.

## 4. Materials and Methods

**Figure 5:**
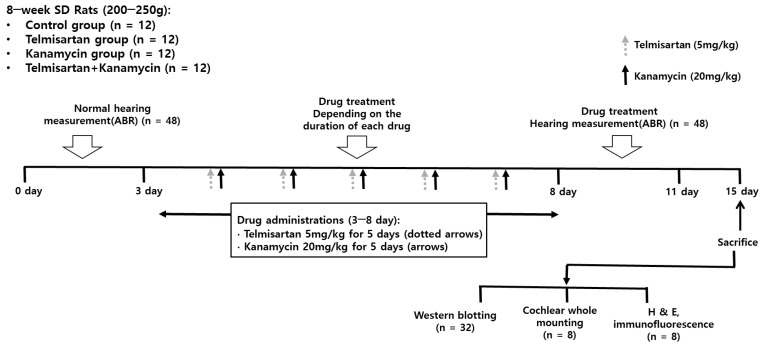
Experimental design of the present study. There were four groups of the control, telmisartan, kanamycin, and telmisartan + kanamycin groups. Hearing levels were measured before and after drug administration.

This study was approved by the Institutional Animal Care and Use Committee of CHA University (IACUC200166). All experimental procedures complied with the regulations of the Institutional Animal Care and Use Committee of CHA University. Forty-eight 8-week-old female Sprague Dawley rats were divided into four groups: control, KM, TM, and KM + TM groups (Figure 5). KM rats were intraperitoneally administered 20 mg/kg/day KM for 5 days. TM rats were intraperitoneally administered 5 mg/kg/day TM for 5 days. KM + TM rats were intraperitoneally administered 20 mg/kg/day KM and 5 mg/kg/day TM for 5 days. Control rats were intraperitoneally administered 50 mL/kg vehicle (normal saline) for 5 days. Pre-treatment (days 0–3) and post-treatment (days 8–11) auditory brainstem response (ABR) thresholds were evaluated. After post-treatment ABR measurements (days 11–15), all rats were euthanized using CO_2_ gas, and the cochleae were harvested.

### 4.1. Auditory Function Tests

The ABR thresholds of both ears at 4, 8, 16, and 32 kHz were measured using the SmartEP system (Intelligent Hearing Systems, Miami, FL, USA) [36]. ABR measurements were conducted under anesthesia by intraperitoneal injection of 40 mg/kg Zoletil and 10 mg/kg xylazine. The reference electrode (vertex), ground electrode (contralateral thigh), and measuring electrode (ipsilateral retroauricular area) were placed. An EC1 electrostatic speaker was fitted to the ipsilateral external auditory canal. The tone bursts (duration, 1562 µs; envelope, Blackman; stimulation rate, 21.2/s; amplitude, 90–20 dB SPL) were applied to the EC1 electrostatic speaker. The ABRs were averaged over 1024 sweeps. The lowest sound amplitude detected in wave III was defined as the ABR threshold.

### 4.2. Cochlear Histologic Examinations

The orientation and presence of the outer hair cells were evaluated by cochlear whole-mount examination. Sixteen cochleae from eight rats (two rats per group) were used for cochlear whole mounts [37]. The dissected cochleae were fixed in 4% paraformaldehyde. The bony labyrinth was decalcified in 120-mM ethylenediaminetetraacetic acid. The membranous labyrinth was discarded, and the cochlear outer hair cell portions were dissected. Blocking and permeabilization were conducted using 1% Triton X-100, 1% bovine serum albumin, and 10% normal goat serum diluted in 10-mM phosphate-buffered saline (PBS) (pH 7.4). Anti-myosin 7A (Santa Cruz, Sc74516) was incubated overnight at 4 °C. After washing with 10-mM PBS (pH 7.4), Alexa 594 anti-mouse IgG (Abcam, ab150108) and 4′,6-diamidino-2-phenylindole dihydrochloride (DAPI) were incubated for 2–3 h at room temperature. After tissue mounting on slides, the slides were examined using a confocal microscope (Zeiss LSM 880, Zeiss, Oberkochen, Land Baden-Württemberg, Germany). Myosin 7a-positive cells were counted and presented as % of loss of outer hair cells. A total of 32 slides (2 slides per cochlea, 8 cochleae per group) were manually counted by two independent researchers.

The morphology of the organ of Corti and spiral ganglial cells was examined using hematoxylin and eosin (H&E) staining [32]. Sixteen cochleae from eight rats (two rats per group) were examined by H&E staining. The cochleae were fixed in 4% paraformaldehyde solution. The bony labyrinth was decalcified. The cochleae were then dipped in a paraffin block. The paraffin block was sectioned to 10-µm thickness. Tissue slides were deparaffinized. The slides were washed three times in ethanol and phosphate-buffered saline. The slides were dipped in hematoxylin for 5 min and eosin for 45 s. The slides were inspected using the EVOS^TM^ XL Core Imaging System (Invitrogen, Carlsbad, CA, USA, #AMEX1000). To visualize spiral ganglial cells, the slides were immunostained with NeuN (Fluor 488 conjugated, ab190195) and DAPI. The immunostained slides were inspected using a confocal microscope (Zeiss LSM 880, Zeiss, Oberkochen, Land Baden-Württemberg, Germany).

### 4.3. Protein Expression Levels of ACE2, HO1, and PPARγ

Sixty-four cochleae from 32 rats (8 rats per group) were used for Western blotting. Cochlear tissues were dipped in protein extraction solution (PRO-PREP^TM^, Intron Technology). After incubation for 30 min on ice, centrifugation was conducted at 13,000 rpm for 5 min. The supernatant was then separated. Purified proteins were quantified using BCA Protein Assay Reagents (Thermo Fisher Scientific, Waltham, MA, USA). Then, the protein samples were subjected to 8% sodium dodecyl sulfate–polyacrylamide gel electrophoresis at 80 V for 90 min. The gels were transferred to polyvinylidene difluoride membranes (Merck Millipore, Burlington, MA, USA) at 300 mA for 90 min. After washing three times with Tris-buffered saline containing Tween-20, the membranes were incubated in blocking buffer (5% nonfat dry milk in Tris-buffered saline containing Tween-20) for 1 h. The membranes were incubated with 1:1000 of anti-ACE2 (Santa Cruz, Sc390851, Dallas, TX, USA), anti-HO1 (Enzo, ADI-SPA-895-F, New York, NY, USA), anti-PPARγ (Abcam, ab272718, Cambridge, UK), and anti-rabbit monoclonal β-actin (Cell Signaling Technology, Danvers, MA, USA, D6A8) overnight at 4 °C. After washing three times, the membranes were incubated with horseradish peroxidase (HRP)-conjugated secondary antibodies (anti-rabbit IgG, HRP-linked; Cell Signaling Technology, #7074S and goat anti-mouse IgG H&L [HRP]; Abcam, #ab97023) for 2 h at room temperature. Protein bands were detected using an enhanced chemiluminescence kit (Bio-Rad, Hercules, CA, USA). The bands were quantified using ImageJ software (National Institutes of Health, Bethesda, MD, USA). The quantified values of each band were normalized to those of β-actin. Fold-changes in protein expression levels were then calculated by comparison with those of the control group.

### 4.4. Statistical Methods

The pre- and post-treatment ABR thresholds were compared using a paired *t*-test for each group. The ABR thresholds and cDNA and protein expression levels were compared between groups using unpaired data. Statistical significance was defined as *p* ≤ 0.05. SPSS version 21.0 (IBM Corp., Armonk, NY, USA) was used for all analyses. All graphs are presented as the means and error bars (± standard deviation (SD)).

## 5. Conclusions

TM protects the cochlea from KM-induced ototoxicity in rat models. The attenuation of oxidative stress involving HO1 and PPARγ was presumed to be linked to the otoprotective effects of TM.

## Data Availability

The data presented in this study are available upon request from the corresponding author.
